# Brusatol inhibits HIF-1 signaling pathway and suppresses glucose uptake under hypoxic conditions in HCT116 cells

**DOI:** 10.1038/srep39123

**Published:** 2016-12-16

**Authors:** Yapeng Lu, Bo Wang, Qian Shi, Xueting Wang, Dang Wang, Li Zhu

**Affiliations:** 1 Institute of Nautical Medicine, Nantong University, Nantong 226019, China; 2 Central laboratory, The first people’s hospital of Huzhou, Huzhou 313000, China

## Abstract

Hypoxia-inducible factor-1 (HIF-1) is an important transcription factor that induces adaptive responses upon low oxygen conditions in human cancers and triggers off a poor prognostic outcome of conventional treatments. In this study, we discovered for the first time that brusatol (BRU), a quassinoid extracted from Brucea Esters, has the capability to inhibit HIF-1 signaling pathway. We found that BRU concentration-dependently down-regulated HIF-1α protein levels under hypoxia or CoCl_2_-induced mimic hypoxia in HCT116 cells without causing significant cytotoxicity. Besides, the transactivation activity of HIF-1 was suppressed by BRU under hypoxic conditions, as well as the expression of HIF-1 target genes, including VEGF, GLUT1, HK2 and LDHA. In addition, BRU can also decrease glucose consumption under hypoxia through inhibition of HIF-1 signaling pathway. Further studies revealed that the inhibitory effect of BRU on HIF-1 signaling pathway might be attributed to promoting degradation of HIF-1α. Interestingly, intracellular reactive oxygen species (ROS) levels and mitochondrial ROS level were both decreased by BRU treatment, indicating the involvment of mitochondrial ROS regulation in the action of BRU. Taken together, these results provided clear evidence for BRU-mediated HIF-1α regulation and suggested its therapeutic potential in colon tumors.

Many malignant and aggressive solid tumors show resistance to conventional therapy due to hypoxic tumor microenvironment^1^. Tumor hypoxia can induce a wide range of biological changes and has been regarded as an important prognostic factor for advanced cancer progression and poor clinical outcome^2^. Hypoxia-inducible factor-1 (HIF-1) is known to trigger adaptive responses of cells under hypoxic conditions through transcriptionally activating hundreds of downstream genes involved in many aspects of cancer development^3^. HIF-1 is a heterodimer consisting of an O_2_-regulated α subunit and a constitutively expressed β subunit^4^, which binds to the consensus sequence the hypoxia-responsive element (HRE) that is always present within HIF-1-regulated genes^5^.

Most of HIF-1-regulated genes are closely associated with tumor development^6^. For example, the genes involved in the metabolic remodeling of cancer cell including PDK1, LDHA, GLUT1, HK2 and microRNA-210, etc. can be directly regulated by HIF-1, promoting cell proliferation^7^,^8^. In addition, activation of HIF-1 pathway promotes tumor angiogenesis, invasion and metastasis, so that patients faced a higher mortality rate and ineffective treatment^9^.

Since HIF-1 is a key regulatory factors in the progress of malignant solid tumors, the inhibition of HIF-1 signaling pathway to the treatment of malignancies has broad clinical application. In recent years, specific inhibitors targeting different steps of HIF-1 signaling pathway, including HIF-1α mRNA expression, HIF-1α protein synthesis, HIF-1α protein stability, HIF-1α/HIF-1β dimerization and HIF-1 transactivation have gained more and more attention on research and development of antitumor agents^6^,^10^,^11^,^12^.

Brusatol (BRU), a quassinoid obtained from Brucea species (Simaroubaceae), is capable of inducing an array of biological responses including antiinflammatory and antileukemic effects in murine models^13^. Recently, BRU was identified as a novel Nrf2 inhibitor by enhancing ubiquitination and degradation of Nrf2 and can sensitize a broad spectrum of cancer cells to chemotherapeutic drugs^14^. In this study, the inhibitory effect of BRU on HIF-1 signaling pathway was identified for the first time, suggesting a therapeutic advantage for the use of BRU in cancer therapy.

## Materials and Methods

### Materials

BRU was obtained from Chengdu pureChem-standard Corp. (Chengdu, China). Dimethyl sulphoxide (DMSO), Trizol, CoCl_2_, Cycloheximide (CHX), MG132, 3-(4,5-dimethyl-2-thiazolyl)-2,5-diphenyl-2-H-tetrazolium bromide (MTT) and 2′,7′-dichlorodihydrofluorescein diacetate (DCFH-DA) were purchased from Sigma Aldrich (St. Louis, MO, US). MitoSOX Red was obtained from Molecular probes (Eugene, OR, USA). HIF-1α monoclonal antibody was purchased from BD Biosciences (San Diego, CA, US). VEGF and β-actin monoclonal antibodies were purchased from Beyotime Corp. (Shanghai, China). The Alexa-Fluor 555 (red)-conjugated secondary antibody was obtained from Invitrogen Corporation (Carlsbad, CA, US). Cell culture reagents were purchased from Gibco (Carlsbad, CA, US). All chemicals were standard analytical grade or higher.

### Cell culture and hypoxic treatment

The human colon cancer cell line HCT116 was obtained from Chinese Academy of Sciences Cell Bank (Shanghai, China). Cells were grown in Dulbecco’s modified eagle medium (DMEM) with 10% fetal bovine serum and 0.5% (v/v) penicillin-streptomycin at 37 °C in a humidified 5% CO_2_ incubator (Thermo Forma Electron Co., Marietta, OH, US). Hypoxia was created by adjusting the environment to 1% O_2_, 5% CO_2_, and 94% N_2_ using a hypoxic work station (Ruskinn Technologies, UK). Mimic hypoxia was created by adding 200 μM CoCl_2_ to medium as previously described^15^.

### MTT assay

The HCT116 cells were seeded into 96-well plates (1 × 10^4^ cells per well). After culturing for 12 h, cells were treated with different concentrations of BRU for 24 h and the viability of the HCT116 cells was analyzed by MTT assay. In brief, cells were stained with 0.5 mg/ml MTT for 4 h at 37 °C. Then the culture medium was removed and MTT formazan crystals were dissolved in 200 μL lysis buffer (20% SDS in 50% N′N-dimethylformamide, pH 4.7). Optical density was measured at a wavelength of 570 nm and background absorbance was subtracted measuring at 690 nm by the use of a microplate reader (Synergy 2^TM^, BioTek, US).

### Trypan blue assay

After treatment, the HCT116 cells were made into a single-cell suspension by trypsin. The suspension was mixed with trypan blue according to the protocol of trypan blue staining kit (Beyotime, Shanghai, China). Invitrogen Countess (HR45, Invitrogen, US) was employed to count the number of viable cells and dead cells. The statistical living cell rate (%) = the number of viable cells/(the number of viable cells and dead cells) ×100%. The data represented the average viability from four separate experiments performed in triplicate.

### PCR analysis

After treatment, total RNA was extracted from the cells with a Trizol reagent, and RNA was reverse transcribed into cDNA with an Omniscript RT kit (Qiagen, Hilden, Germany) according to the manufacturer’s instructions. The primers were synthesized by Sanggon Corporation (Shanghai, China). The primers were as follows: β-actin, 5′-TGA CGG GGT CAC CCA CAC TGT GCC CAT CTA-3′ (forward), 5′-CTA GAA GCA TTG CGG TCG ACG ATG GAG GG-3′ (backward); HIF-1α, 5′-CTC AAA GTC GGA CAG CCT CA-3′ (forward), 5′-CCC TGC AGT AGG TTT CTG CT-3′ (backward); GLUT-1, 5′-ATT GGC TCC GGT ATC GTC AAC-3′ (forward), 5′-GCT CAG ATA GGA CAT CCA GGG TA-3′ (backward); LDHA, 5′-TTG ACC TAC GTG GCT TGG AAG-3′ (forward), 5′-GGT AAC GGA ATC GGG CTG AAT-3′ (backward); and HK2, 5′-TTG ACC AGG AGA TTG ACA TGG G-3′ (forward), 5′-CAA CCG CAT CAG GAC CTC A-3′ (backward). For quantitative real-time reverse transcription PCR (qRT-PCR) analysis, the action mixture consisted of 5 μl SYBR Green, 3 μM each primer, 2 μl cDNA. PCR amplifications were performed on the 7300 real-time PCR system (Applied Biosystems, CA, US). The relative mRNA expression level was calculated by the comparative 2^−ΔΔCt^ method and normalized against β-actin mRNA. For reverse transcription-PCR analysis, amplification was done for 33 cycles, each with denaturation at 95 °C for 30 seconds, annealing at 60 °C for 30 seconds and extension at 72 °C for 30 seconds. The products were analyzed using 1.5% agarose gel electrophoresis. The images were scanned with Tanon ultraviolet imaging system (Tanon-5200Multi, Shanghai, China) and data was analyzed using Image J software. β-actin was used as an internal control.

### Western blot analysis

After treatment, the HCT116 cells were collected and homogenized in a cell lysis buffer consisting of 62.5 mM Tris, pH 6.8, 10 mM DTT, 2% SDS, 10% glycerol, and a protease inhibitor cocktail. Total protein was quantified with the BCA-based protein quantification kit (Thermo Scientific, Rockford, US), and subjected to SDS-poly acrylamide gel electrophoresis. The protein sample was transferred to a PVDF membrane (Millipore, Bedford, MA, US), which was blocked with 5% non-fat dry milk in Tris-buffered saline (TBS, pH 7.4) and incubated with anti-HIF-1α (1:500), anti-VEGF (1:1000), at 4 °C overnight, respectively. After washing with TBST (TBS with 0.1% Tween 20), IRDye 800-conjugated affinity purified goat anti-mouse IgG (1:10000) or goat anti-rabbit IgG (1:10000) was applied at room temperature for 2 h. The images were scanned with ODYSSEY^®^ Infrared Imaging System (LI-COR, Lincoln, Nebraska, US) and data was analyzed with Image J software. β-actin was used as an internal control for normalizing protein loading.

### Luciferase activity assays

Luciferase assay for transcriptional activity of HRE-luciferase was performed as previously reported^16^. In brief, HCT116 cells were grown to 90% confluence, and then plasmid constructs were cotransfected with an internal control vector pRL-TK (Promega, Madiso, WI) (100:1 ratio) to the cells by Lipofectamine 2000 (Invitrogen, Carlsbad, CA). Then cells were treated with different concentrations of BRU for further 12 h and subsequently harvested and luciferase activity was quantitated (Synergy 2^TM^, Biotek, US) using Dual-Luciferase Reporter System (Promega, Madiso, WI).

### Quantification of reactive oxygen species (ROS) levels

The intracellular generation of ROS was also analyzed with the probe DCFH-DA, which is a membrane-permeative fluorescent probe which is widely used to monitor intracellar ROS production^17^. After treatment, cells were incubated with 10 μM H_2_-DCFDA at 37 °C for 30 min. Subsequently, DCF fluorescence distribution of 5 × 10^4^ cells was measured by flow cytometry (Gallios, Beckman Coulter, US). Also, the cells were observed with confocal microscopy (SP8, Leica, Germany) without fixation.

The generation of mitochondrial ROS was analyzed by flow cytometry using the dye MitoSOX Red^18^. In brief, cells were stained with 5 μM MitoSOX Red for 10 min and the fluorescence intensity represents the mitochondrial ROS. Cells were resuspended in 1 ml PBS. The mitochondrial ROS levels in all groups were measured by flow cytometry (Gallios, Beckman Coulter, US).

### Glucose measurement

A total of 4 × 10^5^ cells was plated in 6-well plate and treated with MEM medium containing different concentrations of BRU under hypoxia (1% O_2_). Supernatants were collected after 24 h and glucose consumption was measured using Glucose Assay Kit (Applygen Technologies, Beijing, China) according to the manufacturer’ s indication.

### Statistical analysis

The data are presented as mean ± S.D. Statistical analysis was performed using GraphPad Prism 5 and Microsoft Excel software. Comparisons between multiple groups were analyzed by a one-way analysis of variance (ANOVA) followed by Tukey’s multiple comparison tests as a post hoc comparison. Significant differences between control and BRU-treated samples were determined using two tailed *t*-tests. For all tests, *P* values less than 0.05 were considered statistically significant.

## Results

### BRU inhibits proliferation of HCT116 cells

**Figure 1:**
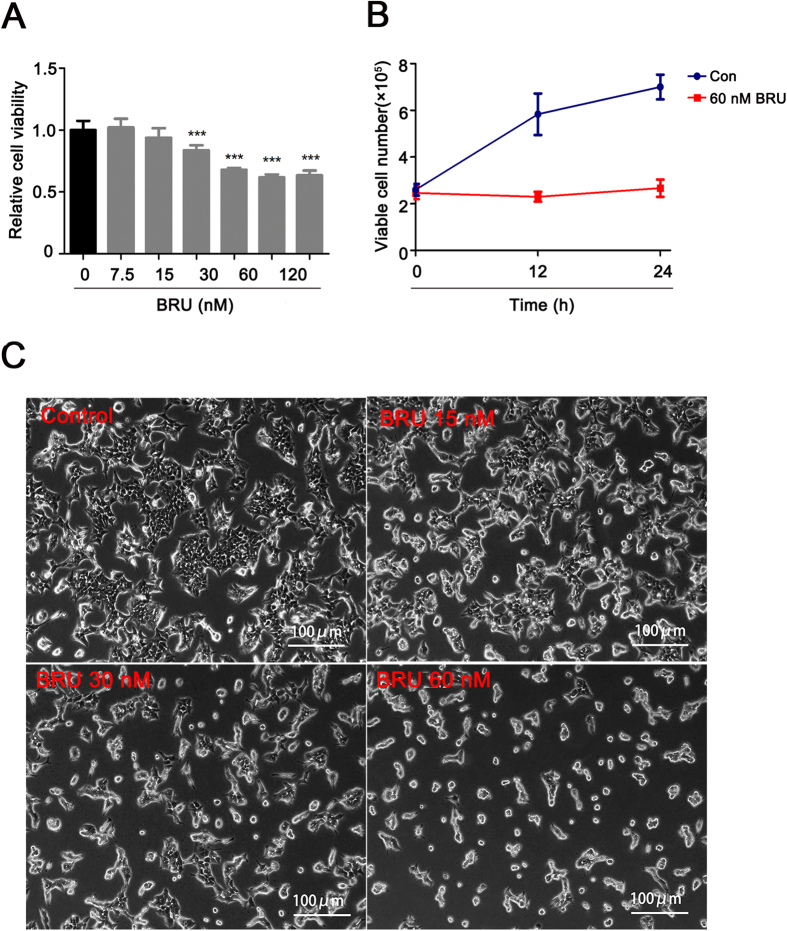
BRU inhibits proliferation of HCT116 cells. (**A**) HCT116 cells were exposed to different concentrations of BRU for 24 h, followed by MTT assay to measure cell viability. Data was presented as means ± S.D. (n = 6). ^***^p < 0.001 versus 0 nM BRU-treated group. (**B**) HCT116 cells were treated with DMSO and BRU (60 nM) for 12 h and 24 h and amount of living cells were detected by trypan blue exclusion assay. (**C**) HCT116 cells were exposed to different concentrations of BRU for 24 h and images were captured under phase contrast microscopy to observe cell morphology. Scale bars, 100 μm.

In order to assess the cytotoxic effect of BRU on HCT116 cells, the cell viability loss induced by various concentrations of BRU was firstly investigated using MTT assay. Results showed that BRU caused the decrease of cell viability when the concentration of BRU is higher than 15 nM (Fig. 1A). BRU caused about 26.7% cell viability loss at 30 nM, and when the concentration of BRU is higher than 60 nM, the cell viability didn’t decrease any more. Then, the effect of BRU on cell cycle in HCT116 cells was examined and the flow cytometric data clearly showed that only S phase was slightly influenced by BRU (Supplementary Figure S1). Besides, the apoptotic sub-G1 phase was not found after the cells were exposed to different concentrations of BRU for 24 h. The anti-proliferation effect of BRU on HCT116 cells was studied using trypan dye exclusion. The data revealed that 60 nM BRU could significantly suppress the proliferation of HCT116 cells within 24 hours without inducing cell death (Fig. 1B). The morphological and quantative change of cells were observed under phase contrast microscopy (Fig. 1C). The control cells exhibited overlapping muti-layer, but BRU-treated cells showed that the interval of cells were expand and overlap reduced. These results together indicated that BRU can effectively inhibit cell proliferation without inducing cell death in HCT116 cells when the concentration is less than 60 nM.

### BRU down-regulates HIF-1α protein levels in hypoxic conditions

**Figure 2:**
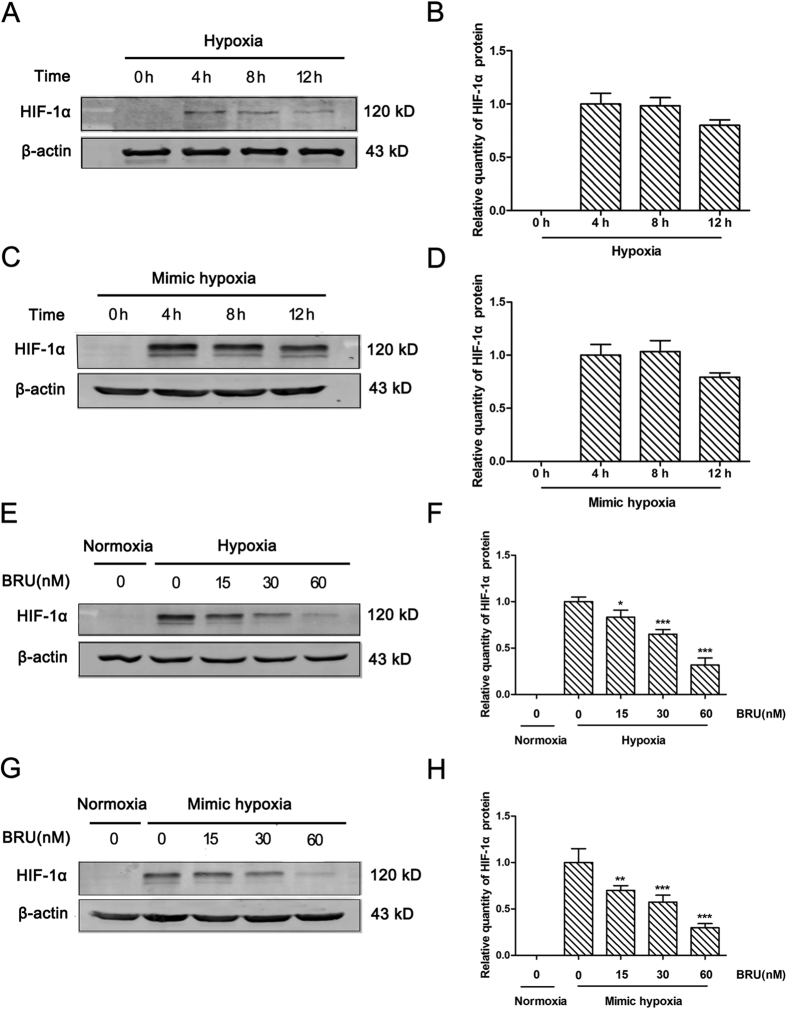
BRU down-regulates HIF-1α protein levels in hypoxic conditions. (**A**) HCT116 cells were treated with hypoxia (1% O_2_) for different time and the quantity of HIF-1α protein was analyzed by Western blot. (**B**) The relative quantity of HIF-1α protein described in (**A**). (**C**) HCT116 cells were treated with 200 μM CoCl_2_-induced mimic hypoxia for different time and the quantity of HIF-1α protein was analyzed by Western blot. (**D**) The relative quantity of HIF-1α protein described in (**C**). (**E**) HCT116 cells were treated with various concentrations of BRU for 4 h under hypoxia (1% O_2_) and the quantity of HIF-1α protein was analyzed by Western blot. (**F**) The relative quantity of HIF-1α protein described in (**E**). Data was presented as means ± S.D. (n = 3). ^***^p < 0.001 and ^*^p < 0.05 versus the hypoxia alone group. (**G**) HCT116 cells were treated with various concentrations of BRU for 4 h under 200 μM CoCl_2_-induced mimic hypoxia and the quantity of HIF-1α protein was analyzed by Western blot. (**H**) The relative quantity of HIF-1α protein described in (**G**). Data was presented as means ± S.D. (n = 3). ^***^p < 0.001 and ^**^p < 0.01 versus the mimic hypoxia alone group.

To characterize the HIF-1α protein expression pattern under hypoxia (1% O_2_) or 200 μM CoCl_2_-induced mimic hypoxia in HCT116 cells, the protein level of HIF-1α was detected by Western blot at different time points. The results showed that the HIF-1α protein levels obviously increased at 4 h under hypoxia or mimic hypoxia in HCT116 (Fig. 2A and B, Fig. 2C and D). Therefore, 4 h of hypoxic treatment was chosen to assess the effect of BRU on HIF-1α protein expression in HCT116 cells. The results showed that BRU down-regulated HIF-1α protein levels in a concentration-dependent manner no matter under hypoxia or mimic hypoxia (Fig. 2E and F,G and H), without affecting the expression of HIF-1β (Supplementary Figure S2). Especially, when the cells were treated with 60 nM BRU, HIF-1α protein was almost undetectable. These data clearly demonstrated that BRU can down-regulate HIF-1α protein levels in hypoxic conditions.

### BRU does not affect the transcriptional level of HIF-1α in hypoxic conditions

**Figure 3:**
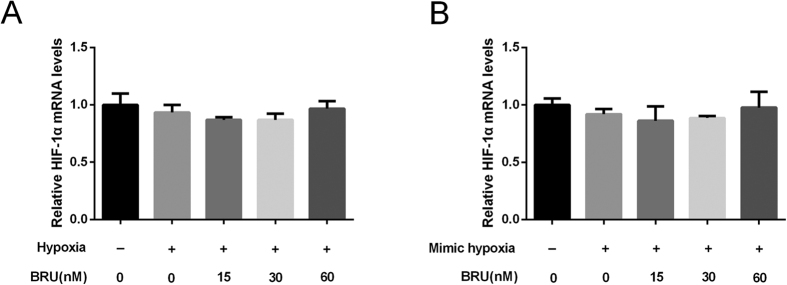
BRU does not affect the transcriptional level of HIF-1α in hypoxic conditions. (**A**) HCT116 cells were cultured under normoxia or hypoxia (1% O_2_) in the presence of various concentrations of BRU for 12 h. The mRNA levels of HIF-1α were analyzed by qRT-PCR and normalized with β-actin mRNA levels. (**B**) HCT116 cells were cultured under normoxia or 200 μM CoCl_2_-induced mimic hypoxia in the presence of various concentrations of BRU for 12 h. The mRNA levels of HIF-1α were analyzed by qRT-PCR and normalized with β-actin mRNA levels.

To determine whether the decrease in HIF-1α protein levels induced by BRU was attributable to a decrease in transcription, we then assessed HIF-1α mRNA levels by qRT-PCR. But the results showed HIF-1α mRNA levels remained unchanged by BRU treatment either under hypoxia or in the presence of CoCl_2_ (Fig. 3A and B), indicating that BRU might be a posttranscriptional regulator of HIF-1α.

### BRU promotes degradation of HIF-1α protein in hypoxic conditions

**Figure 4:**
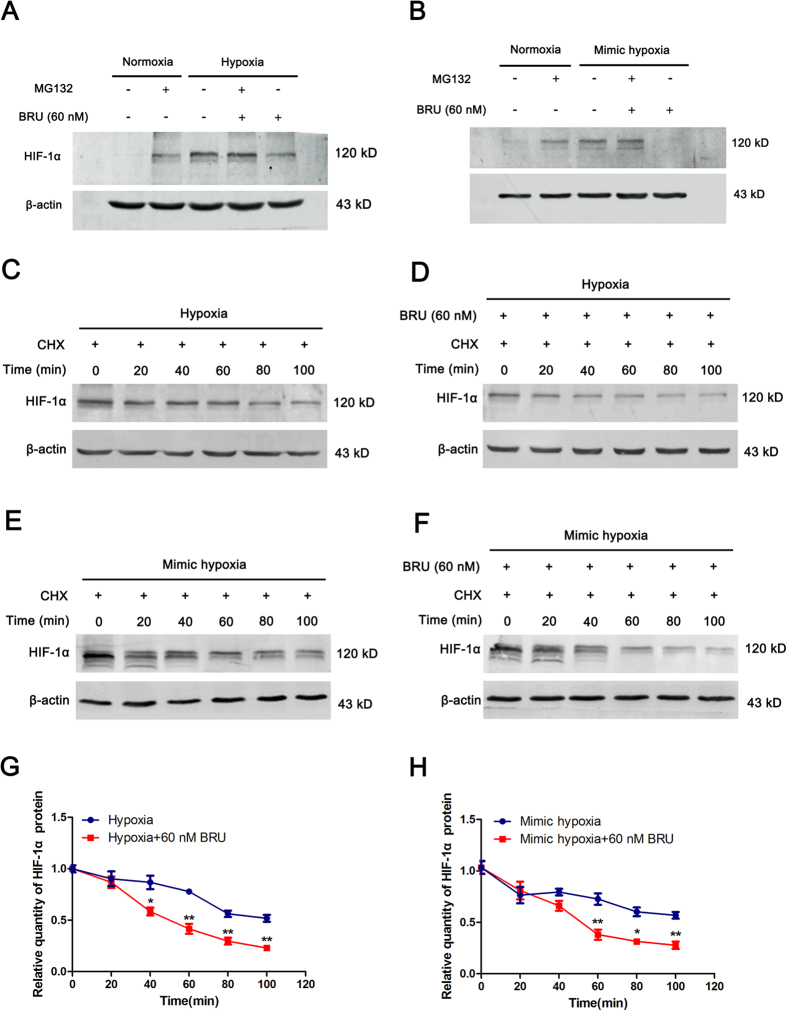
BRU promotes degradation of HIF-1α protein in hypoxic conditions. (**A**) HCT116 cells were cultured under normoxia or hypoxia (1% O_2_) in the presence or absence of 10 μM MG132, with or without 60 nM BRU, for 4 h and the quantity of HIF-1α protein was analyzed by Western blot. (**B**) HCT116 cells were cultured under normoxia or 200 μM CoCl_2_-induced mimic hypoxia in the presence or absence of 10 μM MG132, with or without 60 nM BRU, for 4 h and the quantity of HIF-1α protein was analyzed by Western blot. (**C**) and (**D**) HCT116 cells were firstly incubated with MG132 in normoxia. Then, CHX (50 μg/ml)-containing fresh medium was added into cells, and the cells were further incubated in hypoxia for different time in the presence or absence of 60 nM BRU. At each time point, cells were harvested and the quantity of HIF-1α protein was analyzed by Western blot. (**E**) and (**F**) HCT116 cells were firstly incubated with MG132 in normoxia. Then, CHX (50 μg/ml)-containing fresh medium was added into cells, and the cells were further incubated in CoCl_2_-induced mimic hypoxia for different time in the presence or absence of 60 nM BRU. At each time point, cells were harvested and the quantity of HIF-1α protein was analyzed by Western blot. (**G**) The relative change in the HIF-1α protein levels at each time point described in (**C**) and (**D**). Data was presented as means ± S.D. (n = 3). ^**^p < 0.01 and ^*^p < 0.01 versus the hypoxia alone group. (**H**) The relative change in the HIF-1α protein levels described in (**E**) and (**F**). Data was presented as means ± S.D. (n = 3). ^**^p < 0.01 and ^*^p < 0.01 versus the mimic hypoxia alone group.

To further elucidate the mechanisms underlying the inhibitory effect of BRU on HIF-1α, protein stability of HIF-1α was estimated. Firstly, HCT116 cells were cultured under normoxic or hypoxic conditions for 4 h in the presence or absence of 60 nM BRU, with or without 10 μM MG132, and the quantity of HIF-1α protein was analyzed by Western blot. The results clearly showed that the inhibitory effect of BRU on HIF-1α protein accumulation under hypoxia or CoCl_2_-induced mimic hypoxia were almost reversed by MG132 (Fig. 4A and B), indicating the decreased HIF-1α protein levels induced by BRU under hypoxic conditions is not by inhibiting protein synthesis, but by promoting proteosomal degradation of HIF-1α. Furthermore, the stability of HIF-1α protein under hypoxic conditions in the presence or absence of 60 nM BRU were also investigated. HIF-1α protein accumulation was induced in the presence of 10 μM MG132 in normoxia, and then the protein stability was observed in hypoxic conditions with protein synthesis inhibitor CHX (50 μg/ml) treatment in the presence or absence of 60 nM BRU. The results showed that HIF-1α decreased slowly under hypoxia and about 55% of protein remained at 100 minutes (Fig. 4C and G). However, HIF-1α in BRU-treated cells displayed a more rapid degradation pattern and less than 25% of protein remained at 100 minutes (Fig. 4D and G). Similar results were found in CoCl_2_-induced mimic hypoxic conditions (Fig. 4E,F and H). Taken together, these results clearly indicated that BRU-induced HIF-1α degradation is the leading cause of the impaired HIF-1α response to hypoxia in HCT116 cells.

### BRU inhibits the transactivation function of HIF-1 in hypoxic conditions

**Figure 5:**
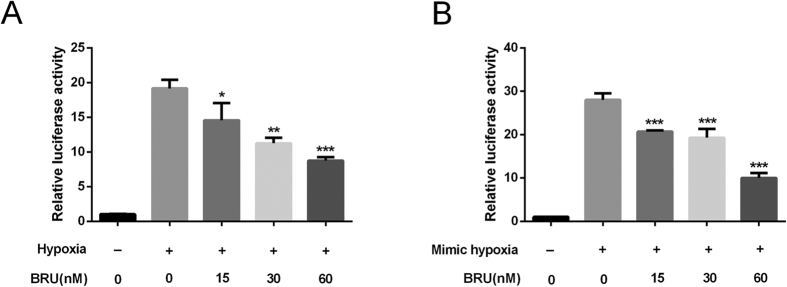
BRU inhibits the transactivation function of HIF-1 in hypoxic conditions. HCT116 cells were transiently cotransfected with the HRE-luciferase plasmid and an internal control vector pRL-TK for 24 h and then treated with or without BRU for 6 h under hypoxia (**A**) or under CoCl_2_-induced mimic hypoxia (**B**), and then luciferase activity was quantitated. Data was presented as means ± S.D. (n = 6). ^***^p < 0.001, ^**^p < 0.01 and ^*^p < 0.05 versus the hypoxia alone group in (**A**). ^***^p < 0.001 versus the mimic hypoxia alone group in (**B**).

As an important transcription factor response to hypoxia, HIF-1 can bind to the promoter region of numerous genes via the HRE and activates the transcription of these target genes. Therefore, we investigated the effect of BRU on transactivation function of HIF-1 by using HRE luciferase reporter assay. The results showed that BRU induced a concentration-dependent reduction in luminescence when compared to untreated controls in both hypoxia and CoCl_2_-induced mimic hypoxia (Fig. 5A and B), indicating the inhibitory effect of BRU on HIF-1 transactivation function in HCT116 cells in hypoxic conditions.

### BRU down-regulates the expression of VEGF in HCT116 cells in hypoxic conditions

**Figure 6:**
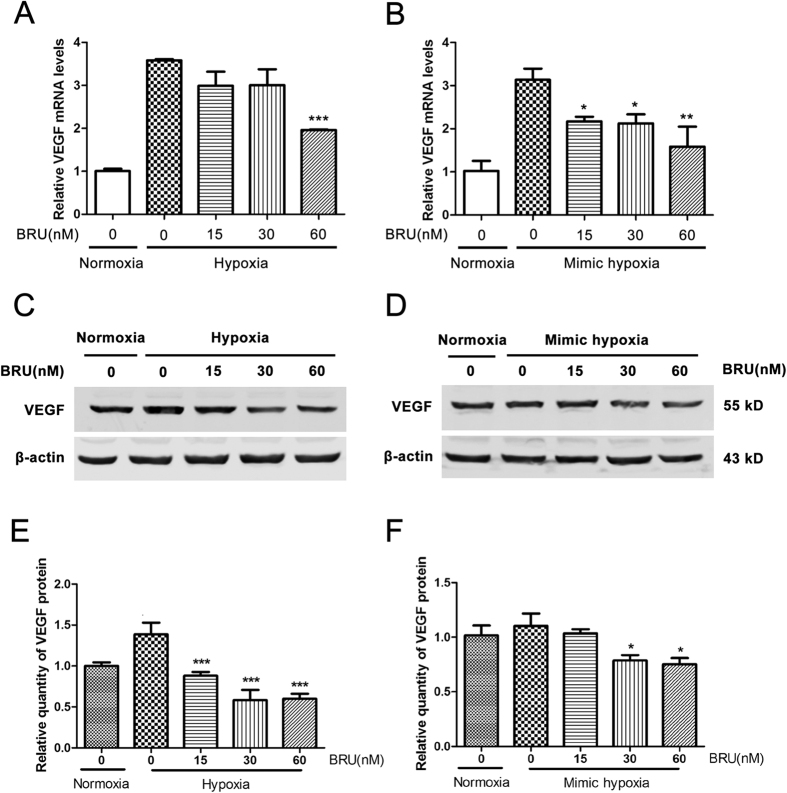
BRU down-regulates the expression of VEGF in HCT116 cells in hypoxic conditions. (**A**) HCT116 cells were treated with various concentrations of BRU for 12 h under normoxia or hypoxia (1% O_2_). The mRNA levels of VEGF were analyzed by qRT-PCR and normalized with β-actin mRNA levels. Data was presented as means ± S.D. (n = 3). ^***^p < 0.001 versus the hypoxia alone group. (**B**) HCT116 cells were treated with various concentrations of BRU for 12 h under 200 μM CoCl_2_-induced mimic hypoxia. The mRNA levels of VEGF were analyzed by qRT-PCR and normalized with β-actin mRNA levels. Data was presented as means ± S.D. (n = 3). ^**^p < 0.01 and ^*^p < 0.05 versus the mimic hypoxia alone group. (**C**) HCT116 cells were treated with various concerntrations of BRU for 24 h under hypoxia and the quantity of VEGF protein was analyzed by Western blot. (**D**) HCT116 cells were treated with various concerntrations of BRU for 24 h under 200 μM CoCl_2_-induced mimic hypoxia and the quantity of VEGF protein was analyzed by Western blot. (**E**) The relative quantity of VEGF protein described in (**C**). Data was presented as means ± S.D. (n = 3). ^***^p < 0.001 versus the hypoxia alone group. (**F**) The relative quantity of VEGF protein described in (**D**). Data was presented as means ± S.D. (n = 3). ^*^p < 0.05 versus the mimic hypoxia alone group.

After the clear inhibitory effect of BRU on HIF-1α protein expression and the HRE reporter gene activity have been proved, the effect of BRU on the expression of VEGF, a well-known downstream target of HIF-1, was also examined under hypoxic conditions. As shown in Fig. 6A and B, treatment with different concentrations of BRU resulted in reduce of VEGF mRNA levels in hypoxia or CoCl_2_-induced mimic hypoxia in a concentration-dependent manner. And we also found that BRU concentration-dependently decreased the protein levels of VEGF under hypoxia and CoCl_2_-induced mimic hypoxia (Fig. 6C and E,D and F).

### BRU inhibits expression of glycolytic enzymes and glucose consumption in HCT116 cells under hypoxia

**Figure 7:**
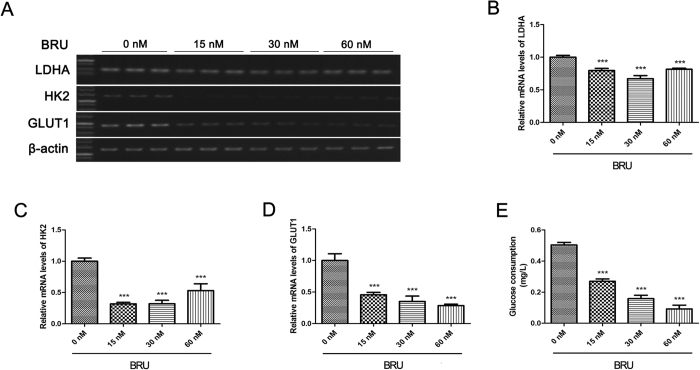
BRU inhibits expression of glycolytic enzymes and glucose consumption in HCT116 cells under hypoxia. (**A**) HCT116 cells were treated with various concentrations of BRU for 12 h under hypoxia (1% O_2_). (**B**), (**C**) and (**D)** The mRNA levels of GLUT1, HK2 and LDHA were analyzed by reverse transcription-PCR and normalized with β-actin mRNA levels. Data was presented as means ± S.D. (n = 3). ^***^p < 0.001 versus the hypoxia alone group (0 nM BRU-treated group). (**E**) HCT116 cells were treated with various concentrations of BRU for 24 h under hypoxia (1% O_2_) and then glucose consumption was measured with commercial kit. Data was presented as means ± S.D. (n = 4). ^***^p < 0.001 versus the hypoxia alone group (0 nM BRU-treated group).

It is generally known that HIF-1 can regulate genes related to glycolysis to reprogram metabolic pathway. Therefore, the effect of BRU on the expression of GLUT1, HK2 and LDHA, the well-known HIF-1 target genes, which are closely related to glycolytic pathway were investigated under hypoxia. The results indicated that BRU can down-regulate GLUT1, LDHA and HK2 mRNA expression in a concentration-dependent manner under hypoxia (Fig. 7A–D). Furthermore, glucose consumption in HCT116 cells under hypoxia decreased in a concentration-dependent manner when treated with BRU for 24 h (Fig. 7E), which suggested that BRU can inhibit glycolysis in HCT116 cells under hypoxia.

### BRU decreases intracellular ROS and mitochondrial ROS levels in HCT116 cells

**Figure 8:**
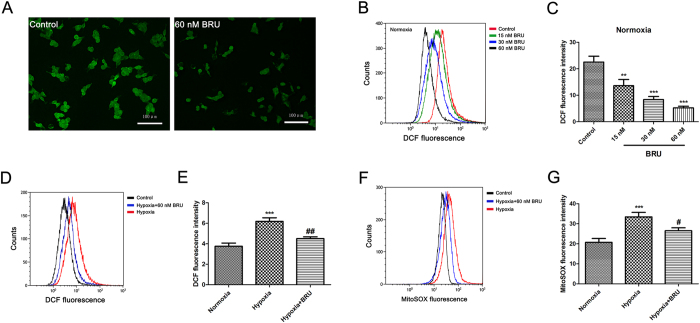
BRU decreases intracellular ROS and mitochondrial ROS levels in HCT116 cells. (**A**) HCT116 cells were treated with 60 nM BRU for 3 h and intracellular ROS generation was analyzed by DCFH-DA using confocal microscope (magnification, 200×). (**B**) HCT116 cells were treated with various concentrations of BRU for 3 h and intracellular ROS generation was analyzed by DCFH-DA using flow cytometry. (**C**) The fluorescence intensity of DCF described in (**B**) was quantitated. Data was presented as means ± S.D. (n = 3). ^***^p < 0.001 and ^**^p < 0.01 versus the control group. (D) HCT116 cells were treated with 60 nM BRU for 3 h under hypoxia (1% O_2_) and intracellular ROS generation was analyzed by DCFH-DA using flow cytometry. (**E**) The fluorescence intensity of DCF described in (**C**) was quantitated. Data was presented as means ± S.D. (n = 3). ^***^p < 0.001 versus the normoxia group, ^##^p < 0.01 versus the hypoxia group. (**F**) HCT116 cells were treated with 60 nM BRU for 3 h under hypoxia (1% O_2_) and mitochondrial ROS generation was analyzed by mitoSOX Red using flow cytometry. (**G**) The fluorescence intensity of mitoSOX described in (**F**) was quantitated. Data was presented as means ± S.D. (n = 3). ^***^p < 0.001 versus the normoxia group, ^#^p < 0.05 versus the hypoxia group.

As shown in Fig. 8A, the intracellular ROS level in HCT116 cells in normoxia was significantly decreased after treated with 60 nM BRU characterized by lower DCF fluorescence. Flow cytometric data also confirmed that BRU dose-dependently inhibited ROS generation in HCT116 cells in normoxia (Fig. 8B and C). In addition, we also found a remarkable decrease in intracellular and mitochondrial ROS production in HCT116 cells when treated with 60 nM BRU under hypoxia (Fig. 8D and E,F and G). Taken together, these results suggested that BRU can inhibit intracellular ROS and mitochondrial ROS generation in HCT116 cells under both normoxia and hypoxia.

## Discussion

It has been reported that BRU, when at micromolar concentrations, can inhibit DNA, RNA, and protein synthesis as well as oxidative phosphorylation in p-388 lymphocytic leukemia cells^19^,^20^. Also, inhibition of overall protein synthesis was observed in some cell lines derived from leukemia, lung adenocarcinoma, Ehrlich carcinoma, and hepatoma when the concentration of BRU is higher than 500 nM^21^,^22^. Recently, BRU has been identified as an unique inhibitor of the Nrf2 pathway at nanomolar concentrations without affecting protein synthesis^14^,^23^, enhancing the anti-tumor efficacy of cisplatin^14^,^24^. In this study, we identified for the first time that BRU has the capability to inhibit HIF-1 signaling pathway. The results showed that BRU inhibited HIF-1α protein accumulation and impaired the activation of its transcriptional targets relating to glucose metabolism and angiogenesis. Furthermore, in search of mechanistic actions of BRU in HIF-1α regulation, we found that HIF-1α protein accumulation during hypoxia was blocked by BRU due to promote HIF-1α protein degradation through proteasomal pathway. It is worth mentioning that no obvious cytotoxicity was observed at the concentrations which were sufficient to inhibit HIF-1 signaling pathway.

As is well known, HIF-1 is the most important inducer of cell adaptation in hypoxia^10^. It is generally reported that HIF-1α is constitutively expressed in many solid tumors^25^,^26^. HIF-1 regulate hundreds of downstream genes, most of which are closely related to tumor growth, metastasis, and invasion^27^, as well as chemoresistance and radioresistance^28^. Targeting HIF-1 has gained more and more attention as an attractive target for cancer therapy over the past several years^6^. A number of different approaches have been proposed to inhibit HIF-1 signaling pathway by targeting different steps of HIF-1α regulation, including HIF-1α mRNA expression, HIF-1α protein synthesis, HIF-1α protein stability, HIF-1α/HIF-1β dimerization, HIF-1 DNA binding, and HIF-1 transactivation^29^.

In the present study, we found BRU can concentration-dependently down-regulate HIF-1α protein levels either in hypoxia or CoCl_2_-induced mimic hypoxia. And no significant change in HIF-1α mRNA levels was found by BRU treatment. It has been proved that the increase of HIF-1α protein during tumor hypoxia is largely attributed to the regulation of HIF-1α stability^30^, which is mediated by the O_2_-dependent regulator PHD and pVHL-26S proteasome^25^. Under hypoxic conditions (<5% O_2_), proline residues of oxygen dependent degradation domain of HIF-1α do not hydroxylate due to the lack of sufficient amount of O_2_, pVHL cannot interact with HIF-1α and finally α monomer remains in cytoplasm and immigrates to nucleus^31^, binding to constantly expressed β monomer and compose HIF-1 transcription factor^32^. As there was no difference in HIF-1α transcript levels, we evaluated the effect of BRU on HIF-1α stability under hypoxic conditions with the proteasome inhibitor MG132 and the protein synthesis inhibitor CHX. The results clearly showed that the inhibitory effect of BRU on HIF-1α protein accumulation under hypoxia or CoCl_2_-induced mimic hypoxia were almost reversed by MG132, indicating the decreased HIF-1α protein levels induced by BRU under hypoxic conditions is not by inhibiting protein synthesis, but by promoting proteosomal degradation of HIF-1α. Unsurprisingly, HIF-1α in BRU-treated cells displayed a more rapid degradation pattern in hypoxic conditions. These results clearly indicated that HIF-1α degradation might be the primary cause of the impaired HIF-1 signaling pathway induced by BRU.

Through suppressing HIF-1α accumulation under hypoxia, BRU may possibly be allowing less stabilization of HIF-1α in the nucleus, which would result in lower downstream genes of HIF-1 expression in cells. Our results confirmed that BRU effectively suppressed the HIF-1 signaling pathway which was characterized by transactivation inhibition and subsequently the down-regulated expression of VEGF, a well known HIF-1 target gene, which is expressed at high levels in several tumors and has been identified as one of the most potent inducers of tumour-associated angiogenesis^33^. In addition, HIF-1 has been validated as the master transcription regulator that orchestrates glycolysis in cancer cells^34^. Unsurprisingly, treatment of HCT116 cells with BRU also resulted in reduction of the expression of glycolysis-related genes such as GLUT1, HK2 and LDHA which are also downstream targets of HIF-1and reduced glucose uptake under hypoxia.

Recently, it has been reported that BRU inhibited the Nrf2 pathway through enhanced ubiquitination and degradation of Nrf2. But the detailed mechanism by which BRU enhances Nrf2 degradation is not clear^14^. In the present study, the results clearly showed that BRU concentration-dependently inhibited ROS generation in HCT116 cells. In addition, a remarkable decrease in intracellular and mitochondrial ROS production was found in HCT116 cells treated with BRU under hypoxia. The decrease in intracellular ROS level by BRU in normoxia may responsible for Nrf2 ubiquitination^35^. Furthermore, it is possible that alterations in cellular ROS levels may affect HIF-1α stability in hypoxia during BRU treatment. The seminal study by Chandel *et al*. has proposed that generation of mitochondrial ROS plays a crucial role in cellular response to hypoxia^36^. Hypoxia increases mitochondrial ROS generation at Complex III, which causes accumulation of HIF-1α protein. These findings reveal that mitochondria-derived ROS are both required and sufficient to initiate HIF-1α stabilization during hypoxia^37^,^38^,^39^,^40^. The decrease of ROS levels by BRU may restore cellular Fe^2+^, which is required for PHD activity, through its oxidation into Fe^3+^, thereby activating PHDs and leading to the degradation of HIF-1α^36^,^41^. Mitochondria is the one of the most important sources of ROS^38^. But we found BRU has no obvious effect on mitochondrial function, such as ATP generation and mitochondrial membrane potential (data not shown). The detailed mechanism by which BRU affects intracellular and mitochondrial ROS level warrants further investigation.

In summary, our results clearly demonstrated that BRU inhibited HIF-1 signaling pathway in hypoxia through promoting degradation of HIF-1α, which subsequently inhibited the expression of the HIF-1 target genes and disturbed metabolic reprogramming of HCT116 cells under hypoxia. The inhibitory effect of BRU on HIF-1 signaling pathway might be dependent on the capacity of BRU to block intracellular ROS, which then downregulated HIF-1α accumulation by activating PHDs. These results may be translatable to clinical treatment modalities because hypoxic microenvironments within solid tumors correlate with tumor metastasis and resistance to drug and radiation treatments.

## Additional Information

**How to cite this article**: Lu, Y. *et al*. Brusatol inhibits HIF-1 signaling pathway and suppresses glucose uptake under hypoxic conditions in HCT116 cells. *Sci. Rep.* **6**, 39123; doi: 10.1038/srep39123 (2016).

**Publisher's note:** Springer Nature remains neutral with regard to jurisdictional claims in published maps and institutional affiliations.

## Supplementary Material

Supplementary Information
