# Supplementary material for: Brusatol inhibits HIF-1 signaling pathway and suppresses glucose uptake under hypoxic conditions in HCT116 cells
Source: Sci Rep. 2016 Dec 16;6:39123. doi: 10.1038/srep39123 (PMC5159874; doi:10.1038/srep39123)

Brusatol inhibits HIF-1 signaling pathway and suppresses glucose uptake under hypoxic conditions in HCT116 cells

Yapeng Lu[1], Bo Wang[1], Qian Shi[2], Xueting Wang[1], Dang Wang[1], Li Zhu[1,*]

[1] Institute of Nautical Medicine, Nantong University, Nantong 226019, China

[2] Central laboratory, The first people's hospital of Huzhou, Huzhou 313000, China

Supplementary information

Supplementary figure 1

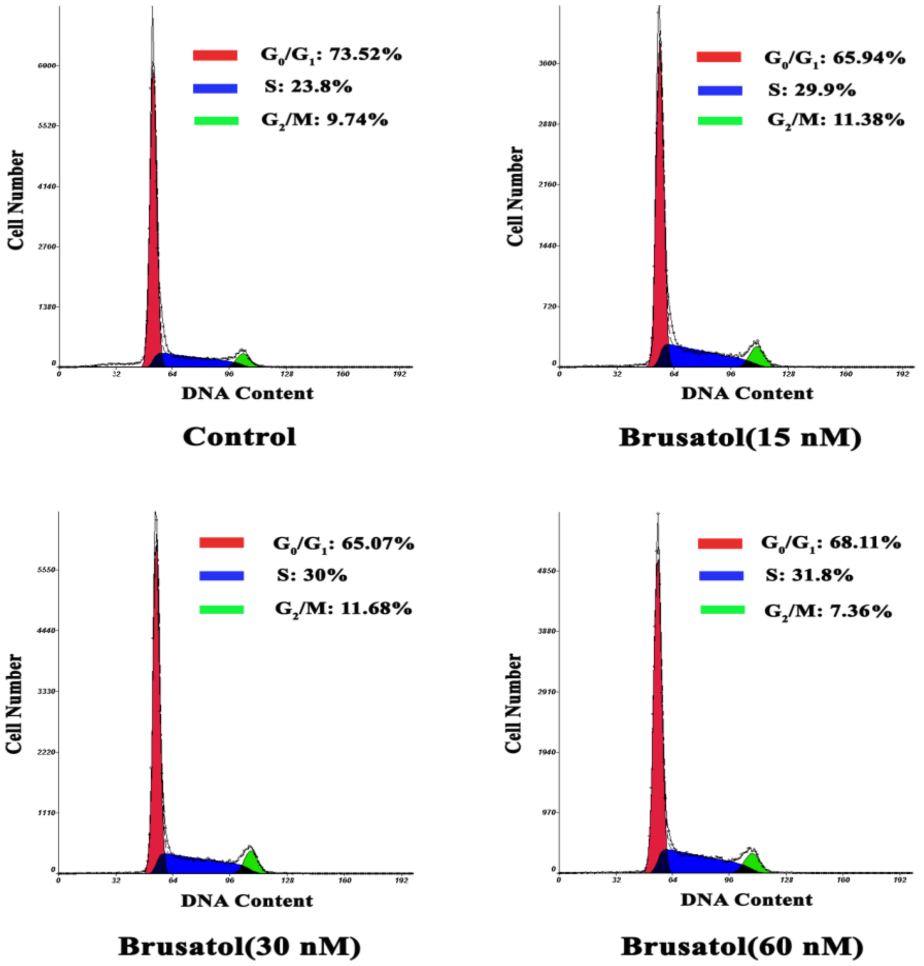

Supplementary figure 1: The effect of BRU on cell cycle in HCT116 cells. HCT116 cells were treated with BRU (15, 30, 60 nM) for 24 h, cell cycle distribution was detected by flow cytometry.

Supplementary figure 2

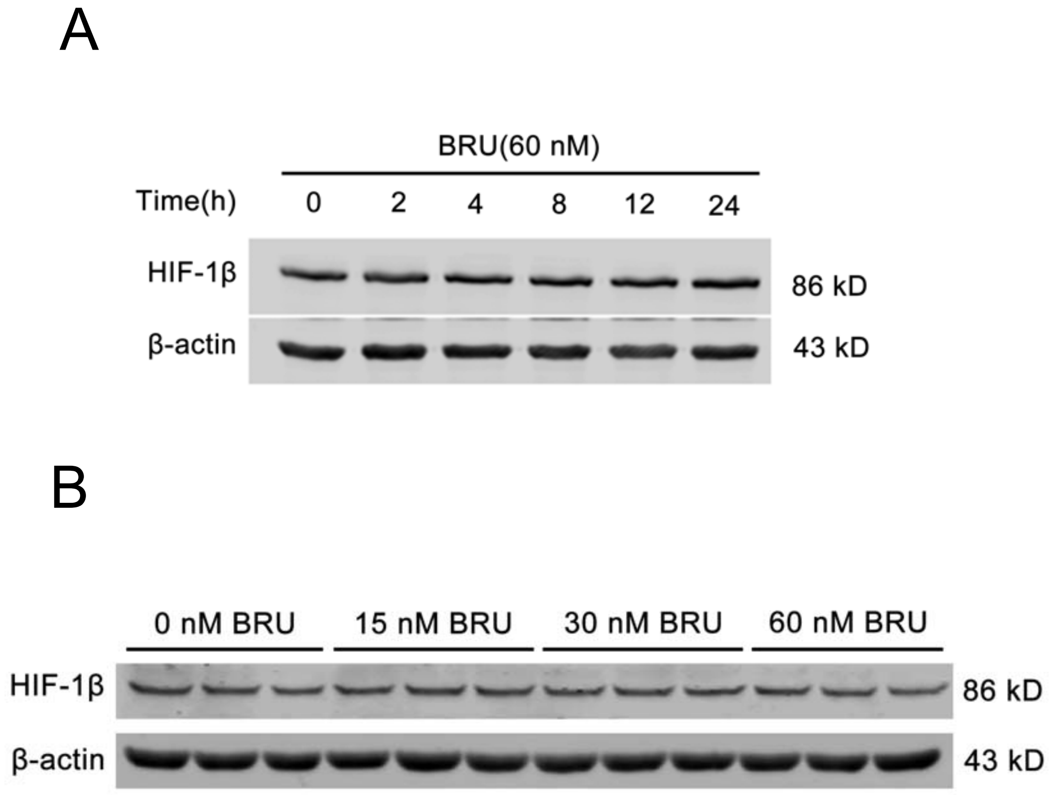

Supplementary figure 2: BRU does not affect the expression level of HIF-1β in HCT116 cells in hypoxic conditions. (A) HCT116 cells were treated with 60 nM BRU for different time under hypoxia (1% O_2) and the quantity of HIF-1α protein was analyzed by Western blot. (B) HCT116 cells were treated with various concentrations of BRU for 4 h under hypoxia (1% O_2) and the quantity of HIF-1α protein was analyzed by Western blot.

The figures of full length blots

Full-length gels and blots for figure 2

Fig 2A

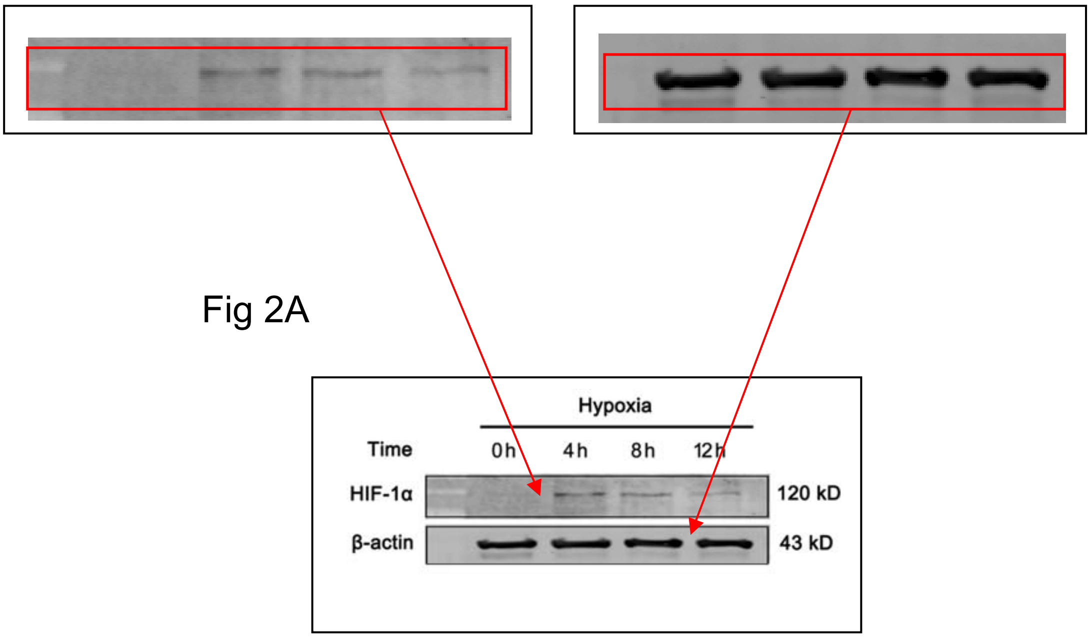

Fig 2C

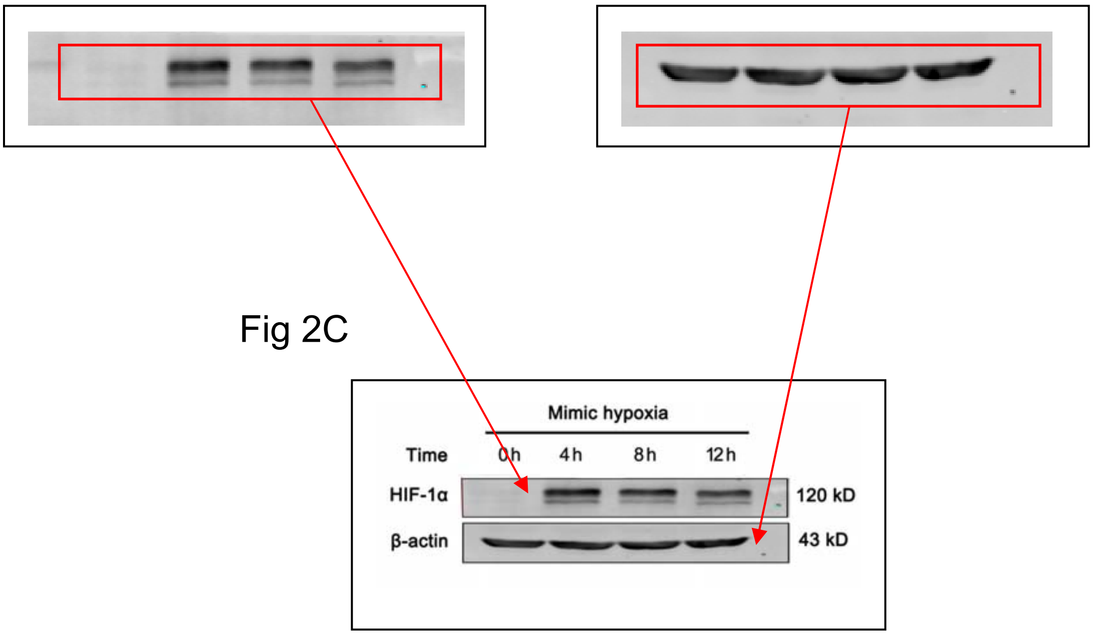

Fig 2E

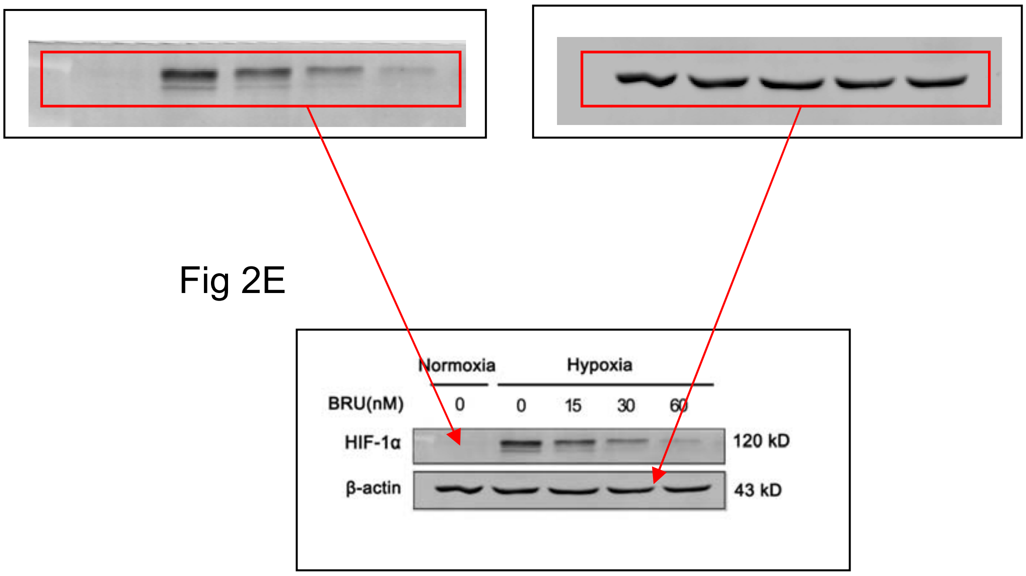
Normoxia
Hypoxia
BRU(nM)
0
0
15
30
60
HIF-1α
120 kD
β-actin
43 kD

Fig 2G

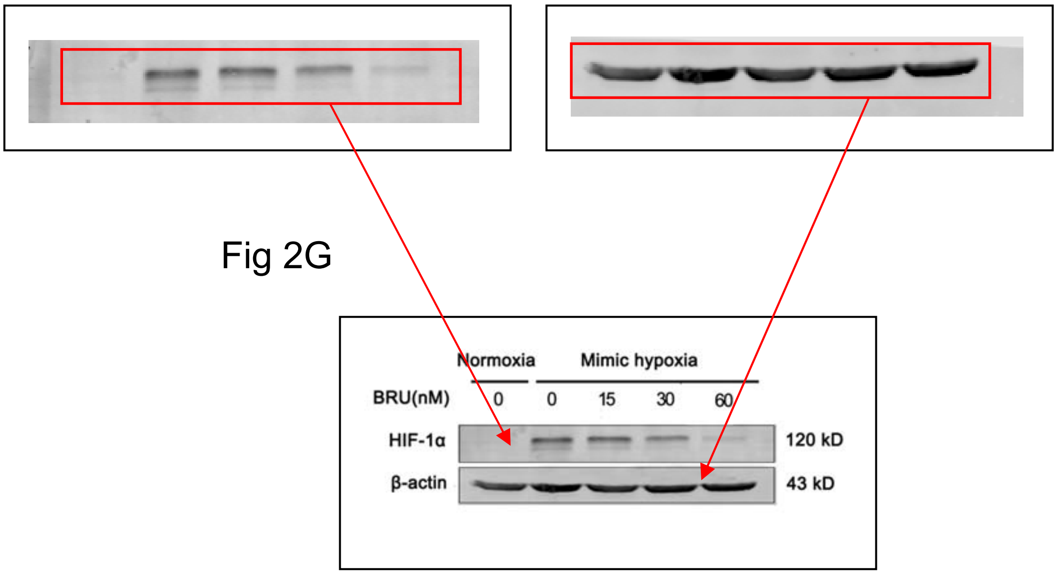
Normoxia
Mimic hypoxia
BRU(nM)
0
0
15
30
60
HIF-1α
120 kD
β-actin
43 kD

Full-length gels and blots for figure 4

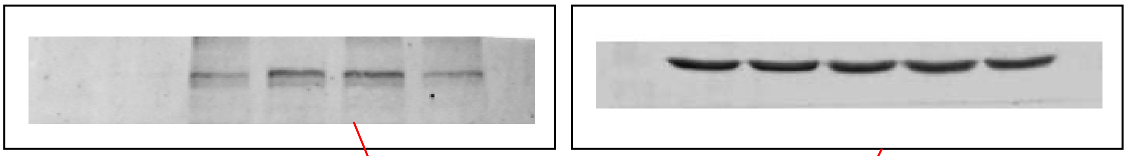

Fig 4A

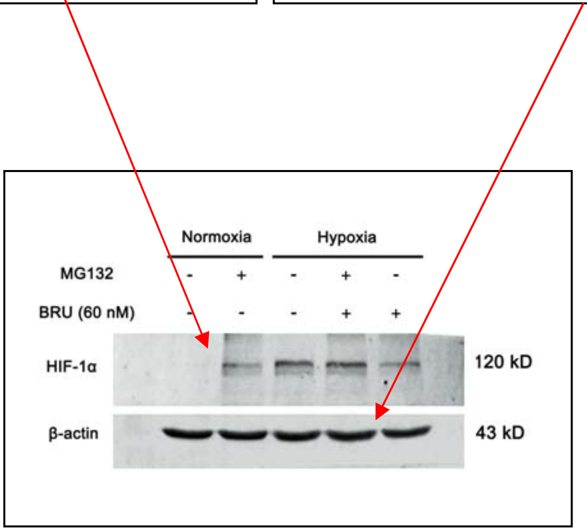

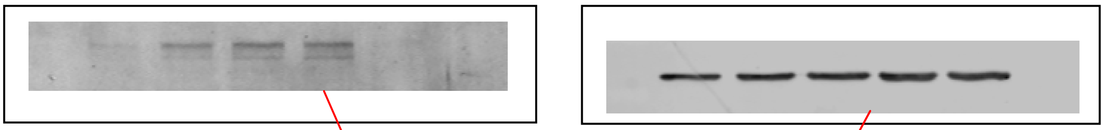

Fig 4B

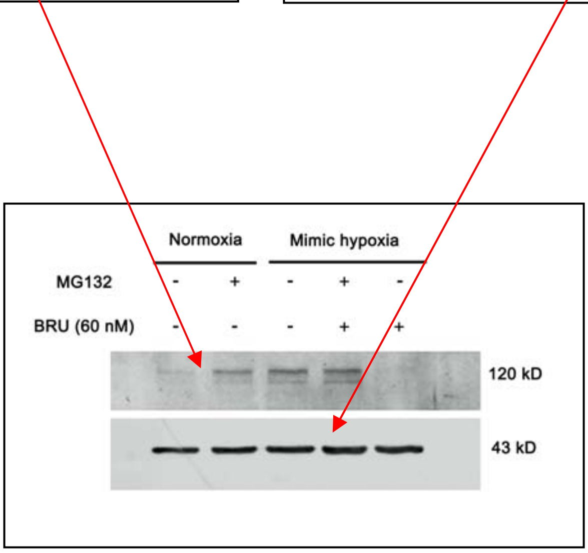

Fig 4C

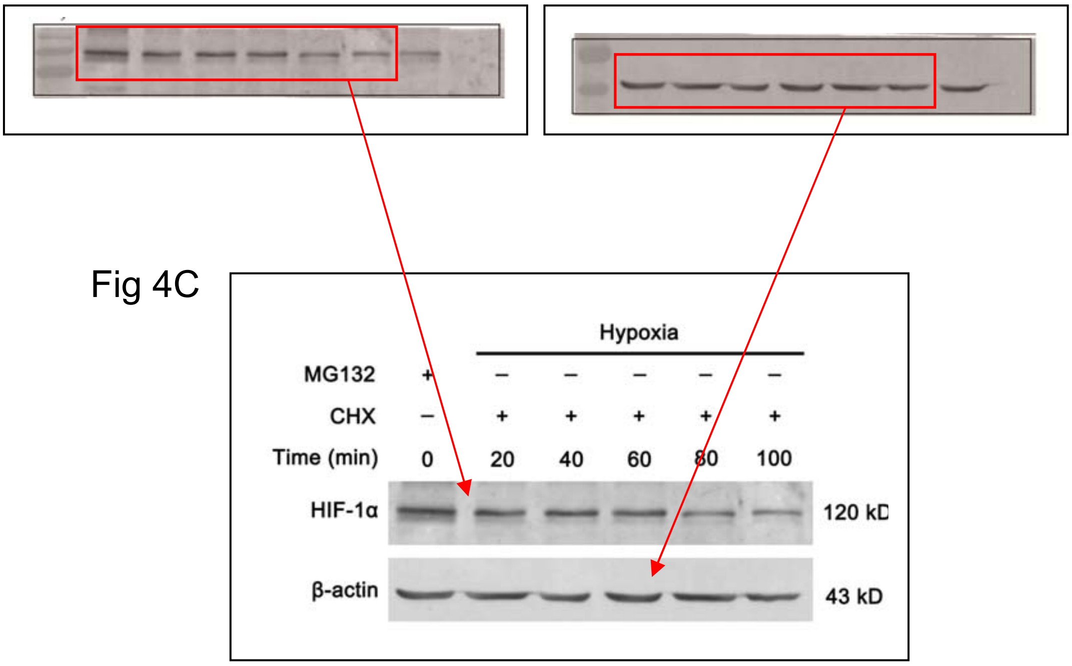

Hypoxia
MG132 + – – – – –
CHX – + + + + +
Time (min) 0 20 40 60 80 100
HIF-1α
120 kD
β-actin
43 kD

Fig 4D

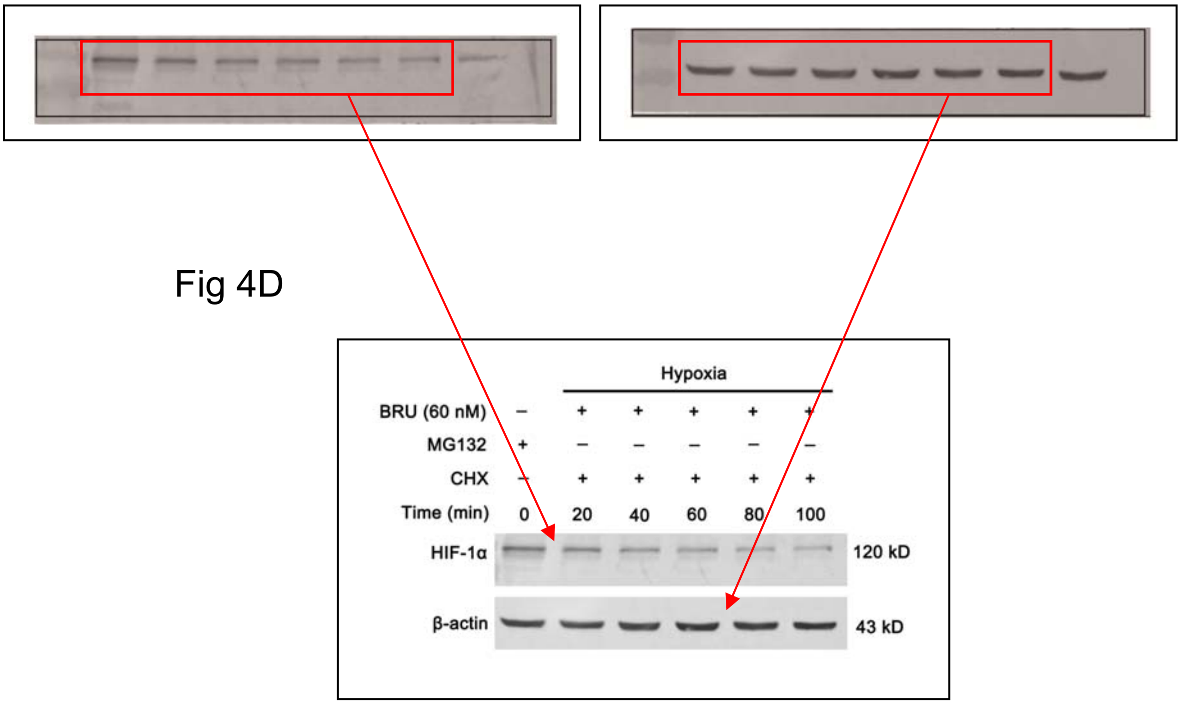

Hypoxia
BRU (60 nM) – + + + + +
MG132 + – – – – –
CHX – + + + + +
Time (min) 0 20 40 60 80 100
HIF-1α
120 kD
β-actin
43 kD

Fig 4E

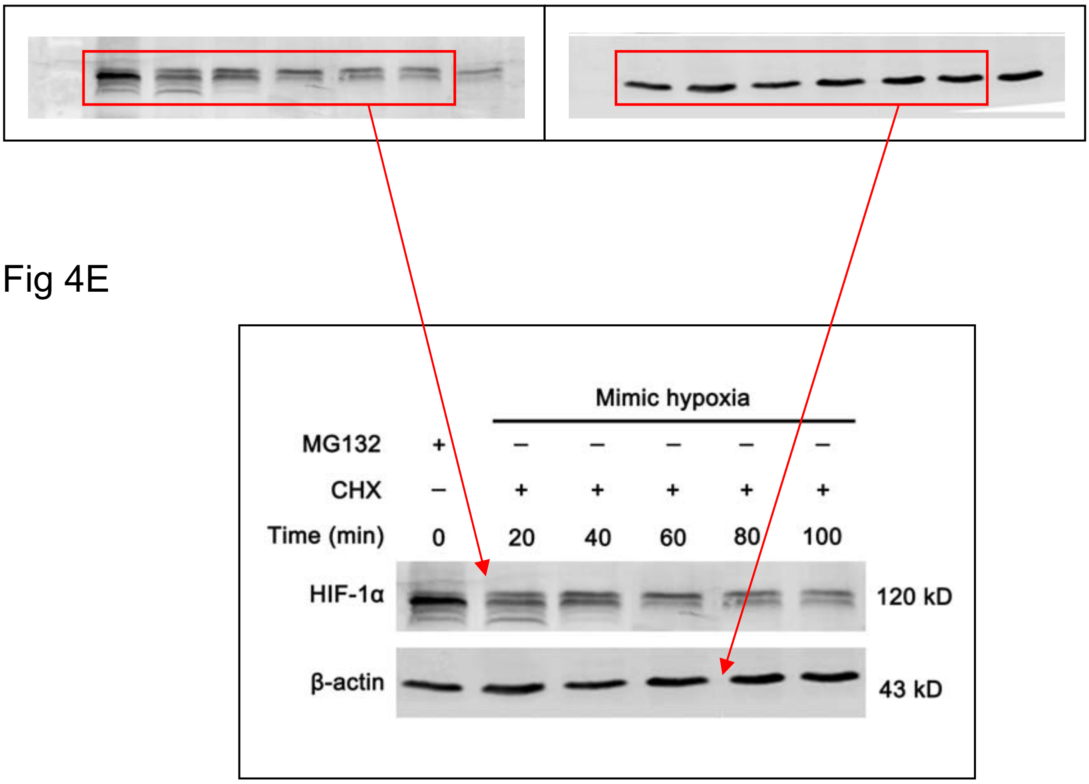
Mimic hypoxia
MG132 + – – – – –
CHX – + + + + +
Time (min) 0 20 40 60 80 100
HIF-1α
120 kD
β-actin
43 kD

Fig 4E

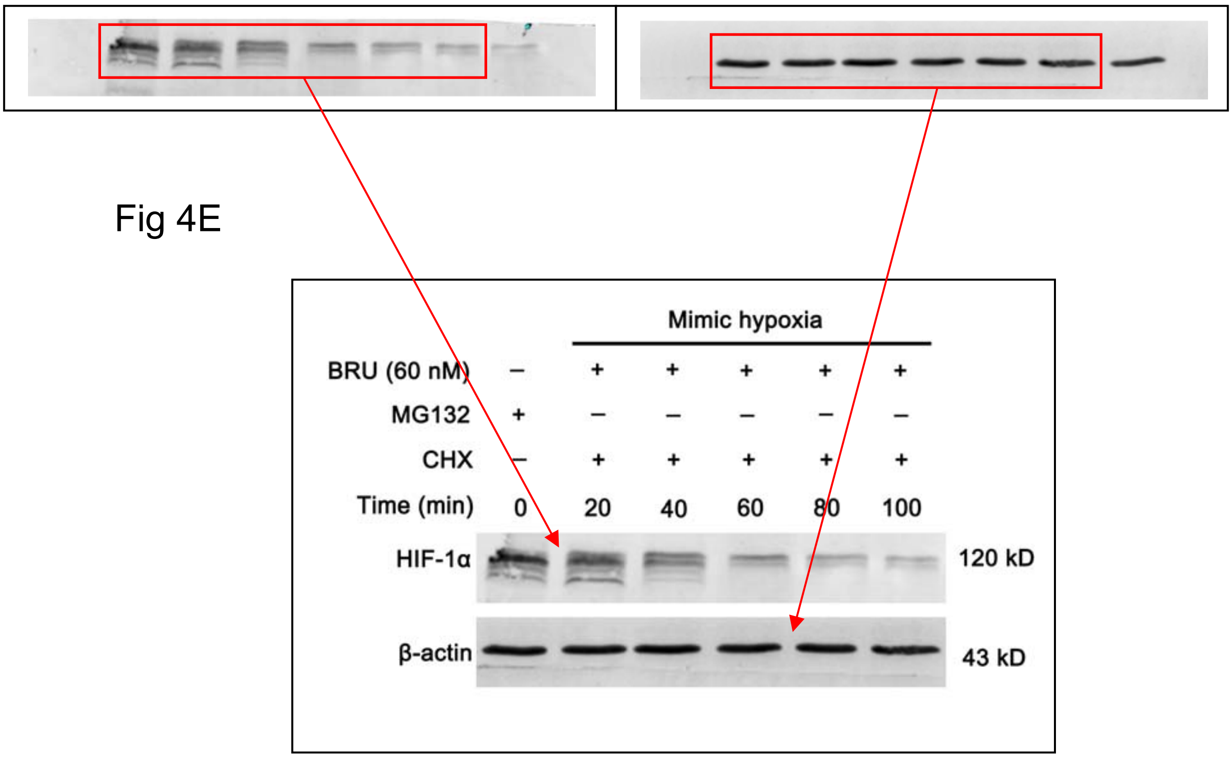
Mimic hypoxia
BRU (60 nM) – + + + + +
MG132 + – – – – –
CHX – + + + + +
Time (min) 0 20 40 60 80 100
HIF-1α
120 kD
β-actin
43 kD

Full-length gels and blots for figure 6

Fig 6C

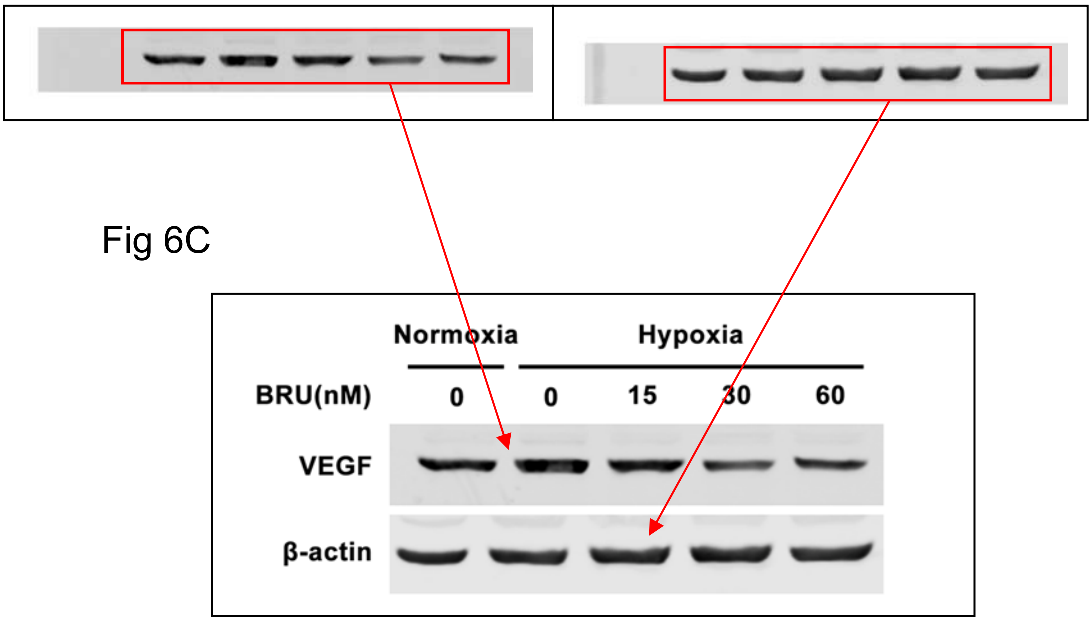

Fig 6D

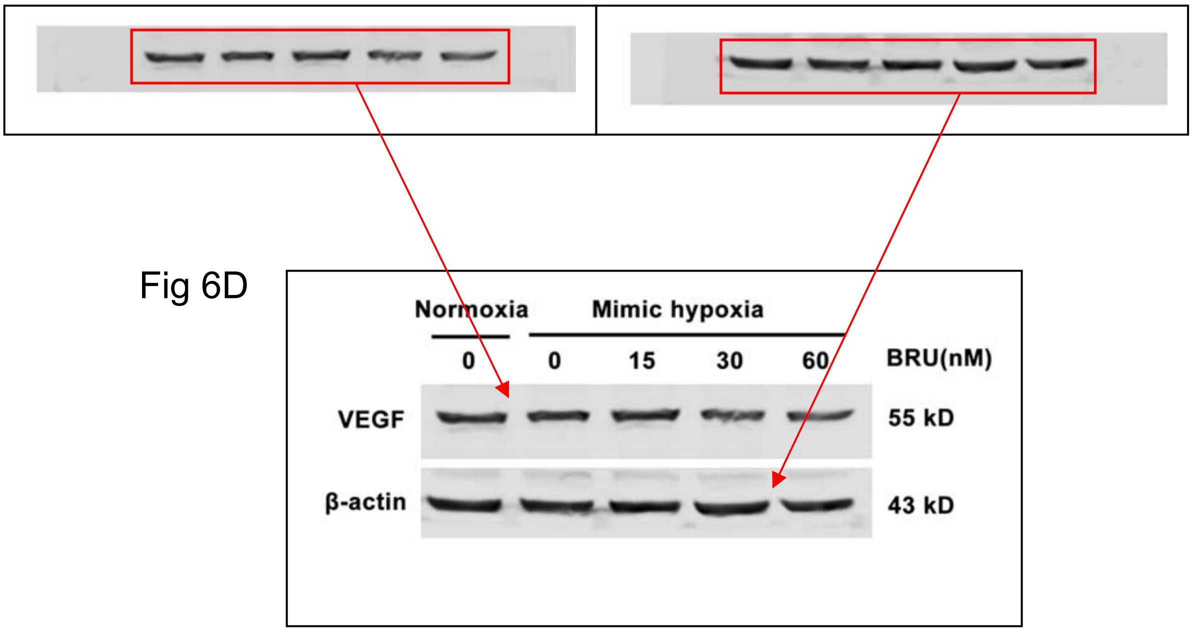

Full-length gels and blots for figure 7

Fig 7A

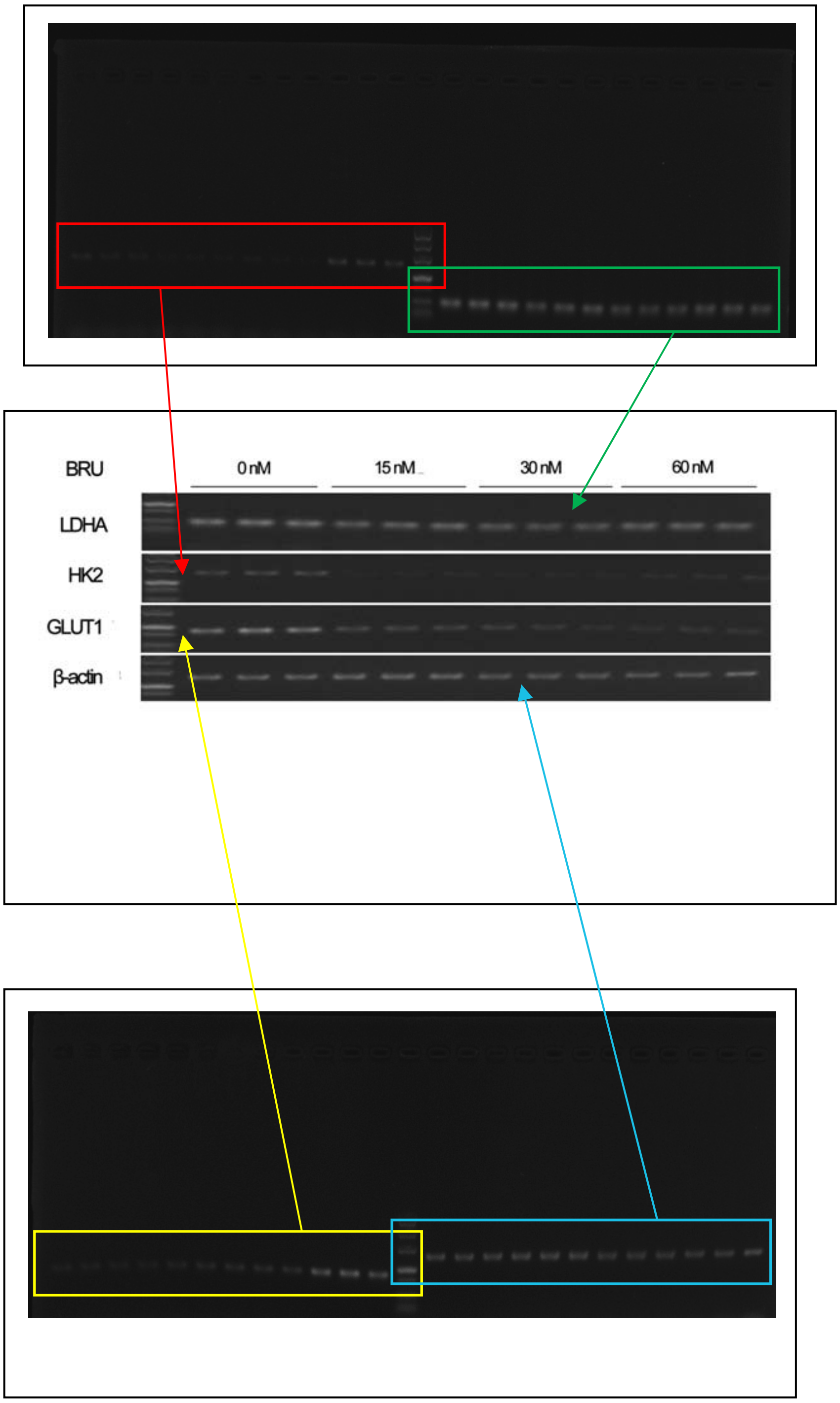

Supplement: Supplementary Information [file srep39123-s1.pdf]
